# Neural circuits provide insights into reward and aversion

**DOI:** 10.3389/fncir.2022.1002485

**Published:** 2022-10-28

**Authors:** Wanqi Chen

**Affiliations:** School of Psychology, Shenzhen University, Shenzhen, China

**Keywords:** reward, aversion, ventral tegmental area, nucleus accumbens, basal forebrain

## Abstract

Maladaptive changes in the neural circuits associated with reward and aversion result in some common symptoms, such as drug addiction, anxiety, and depression. Historically, the study of these circuits has been hampered by technical limitations. In recent years, however, much progress has been made in understanding the neural mechanisms of reward and aversion owing to the development of technologies such as cell type-specific electrophysiology, neuronal tracing, and behavioral manipulation based on optogenetics. The aim of this paper is to summarize the latest findings on the mechanisms of the neural circuits associated with reward and aversion in a review of previous studies with a focus on the ventral tegmental area (VTA), nucleus accumbens (NAc), and basal forebrain (BF). These findings may inform efforts to prevent and treat mental illnesses associated with dysfunctions of the brain’s reward and aversion system.

## Introduction

As utilitarian jurists emphasize, the basis of law is not the text on paper but the concrete behavioral expectations of reward and aversion. In this sense, reward—that is, the preference for obtaining resources necessary for survival and evolution—and aversion—that is, the desire to escape punishments in the form of conditions not conducive to survival—have long been considered significant forces in guiding human behaviors in accordance with Darwin’s theory of evolution. Thus, the human nervous system has developed distinct functional circuits associated with reward and aversion, the dysfunction of which has been shown to correlate strongly with mental illnesses such as anxiety and depression (Dockstader and van der Kooy, 2001; Malkesman et al., 2005; Morgan et al., 2013).

A reward induces pleasant feelings, generates appetitive and consummatory behaviors, and, eventually, reinforces certain behaviors (Bromberg-Martin et al., 2010). Rewards are essential for the survival of individuals and, therefore, their genes, supporting such basic physiological processes as eating and reproduction. For example, consummatory behavior, as a behavioral model, responds to stimuli and achieves a certain motivational satisfaction. Predictive stimuli can also induce appetitive or approach behavior in response to rewards.

**FIGURE 1:**
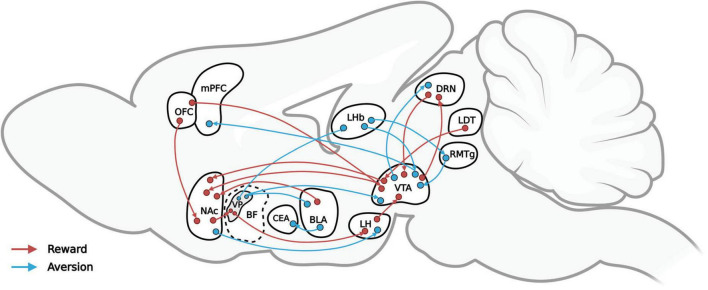
A simplified representation of the reward-mediating (red) and aversion-mediating (blue) neural pathways that recent optogenetics-based behavioral studies have verified.

Conversely, aversive stimuli, or punishments, can trigger negative emotions (including disgust, anxiety, and fear), thereby leading to avoidance and decreasing the likelihood of similar behavioral expressions. Aversive stimuli may cause pain or discomfort and are usually associated with biologically harmful or destructive circumstances or events, such as extreme heat or cold, bitter tastes, electric shock, and loud noises as well as verbal warnings, gestures, and so on. Breakthroughs in the neural mechanisms of reward modulation have been few in the absence of means to modulate specific projections precisely, but recent technological advances, such as the neuropixels and neuronal tracing technique as well as optogenetics, are making greater precision possible. This review, as Figure 1 shows, by integrating the results of previous studies, maps the reward-mediating neural pathways (in red) and the aversion-mediating neural pathways (in blue) that recent optogenetics-based behavioral studies have identified. The nucleus accumbens (NAc), basal forebrain (BF), ventral tegmental area (VTA), and lateral hypothalamus (LH) are among the structures involved in the regulation of reward. Among them, the dopamine (DA) neurons in VTAs emit various reward signals according to their input and output circuits that help to regulate reward-guided behavior (Lammel et al., 2011, 2012). In particular, the dopamine receptor-1 (D1) neurons and dopamine receptor-2 (D2) neurons of the NAc are involved in the regulation of reward, drug addiction, and motivation (Soares-Cunha et al., 2016, 2020; Smith et al., 2017). The activation of dynorphin neurons in the NAc overlaps for the most part that of D1 receptors, resulting in aversion. The projection from D1 medium spiny neurons (MSNs) project to the LH regulates aversion-guided behavior (O’Connor et al., 2015; Massaly et al., 2019; Pignatelli et al., 2021). The ventral pallidum (VP) area of the BF is considered the “common pathway” for the basal ganglia (Smith et al., 2009), and the excitatory and inhibitory neurons in the BF play important roles in the regulation of reward and aversion (Faget et al., 2018; Tooley et al., 2018).

This review connects what is known about the neural regulatory circuits involved in reward and aversion based on the latest research in the field of neuroscience with potential clinical implications for the treatment of addiction, anxiety, depression, and other neuropsychiatric diseases.

## Role of the ventral tegmental area and nucleus accumbens in reward and aversion

### Ventral tegmental area

The VTA is an area of the midbrain dopamine reward system located in the midbrain area between the substantia nigra and the rubrum (Corbett and Wise, 1980). It is a heterogeneous and highly complex structure with multiple types of neurons, including neurons that release GABA and glutamate, though the behavioral impact of VTA activity has long been considered to be driven exclusively by dopaminergic output (Yamaguchi et al., 2007). VTA glutamate neurons selectively express vesicular glutamate transporter type 2 (VGLUT2) mRNA and are found mainly in the medial VTA, representing approximately 2–3% of the total VTA cell population (Nair-Roberts et al., 2008). These neurons project to several areas in the brain, including the NAc, LH, VP, amygdala, and medial prefrontal cortex (mPFC) (Yamaguchi et al., 2011; Hnasko et al., 2012). Approximately 20–40% of VTA cells are GABAergic (Carr and Sesack, 2000; Nair-Roberts et al., 2008; Margolis et al., 2012) and project to several forebrain regions (Fields et al., 2007). It has been reported that the *in vivo* optogenetic activation of VTA GABA neurons in adult mice disrupts reward consumption while activation of VTA GABAergic projections to the NAc results in the detectable release of GABA but does not alter reward consumption (van Zessen et al., 2012).

Ventral tegmental area is the main source of dopamine in the midbrain reward system. VTA DA neurons serve a central role in regulating motivation, reward, and other behaviors (Wise, 1989, 2004; Berridge and Kringelbach, 2015; Pignatelli and Bonci, 2015; Schultz, 2016; Watabe-Uchida et al., 2017). The VTA sends projections to the NAc and also integrates glutamate input from areas such as the PFC, amygdala, and hippocampus (HIP) (Liu et al., 2008). Studies of related damage, receptor blockade, and drug addiction have shown that DA neurons play an important role in reward circuits. A blockade of DA receptors or damage to DA neurons reduces the positive effects of intracranial self-stimulation (ICSS) and the addictive effect of drugs such as cocaine and amphetamines (Fouriezos and Wise, 1976; Wise, 2006). Input-output analyses using TRIO and cTRIO techniques (Schwarz et al., 2015) developed in recent years enable dissection of the VTA DA circuits according to their output targets (Beier et al., 2015). Lammel et al. (2011, 2012) demonstrated that VTA DA neurons signal various reward values on the basis of their input and output circuits. Using optogenetics, researchers can selectively excite or inhibit the VTA neurons. The pathway from the LDT to the VTA DA to the NAc (NAcLat) signals a reward and that from the lateral habenula (LHb) to the VTA DA to the mPFC signals aversion (Nieh et al., 2013).

The reward prediction error (RPE) hypothesis provides a simple account of the role of DA in the learning of rewards and the behaviors associated with them. Cai et al. (2020) recorded and manipulated VTA DA neurons in mice during fear extinction, in which process, their fiber photometry recordings showed, the DA neurons in the medial and lateral VTA displayed distinct activity profiles. Further, the medial VTA activity more closely reflected the RPE while the lateral VTA activity more closely reflected a salience-like signal. This research showed that distinct signals in the medial and lateral VTA dopamine neurons modulate fear extinction at various times. Moreover, in a study applying machine learning to analyze the relationship between the VTA DA and RPE, (Lowet et al., 2020) suggested that the combined activity of VTA DA neurons encodes not just the mean but rather the complete distribution of reward through an expectable code. Meanwhile, (Dabney et al., 2020) reported on distributional reinforcement learning in the brain and also found a distributional code for value in DA-based reinforcement learning, thereby providing strong evidence for a neural realization of distributional reinforcement learning.

Despite the robustness of the RPE account of DA signals, some researchers have challenged this “canonical” view. Thus, several studies have reported that dopamine neurons are activated by aversive stimuli in addition to reward stimuli (Matsumoto and Hikosaka, 2009). To explain the functional diversity of DA, (Matsumoto and Hikosaka, 2009; Bromberg-Martin et al., 2010) argued that DA neurons of one type encode the motivational value, being excited by reward stimuli and inhibited by aversive events, and support the brain’s systems for seeking goals, evaluating outcomes, and learning value. DA neurons of another type encode motivational salience, being excited by both rewards and aversive stimuli, and support the brain’s systems for orienting, cognitive processing, and motivation. In addition to their value- and salience-coding activities, both types of DA neurons transmit alerting signals. Moreover, these two populations of DA neurons are spatially separated, with the former being distributed mainly in the dorsolateral substantia nigra compact area (SNc) and the latter in the ventromedial SNc and lateral VTA. An unresolved question concerns the proportion of DA neurons excited by aversive stimuli and whether they are the specific subpopulation that projects to specific targets. Notably, a recent study in mice found that roughly equivalent numbers of putative VTA DA neurons are activated and inhibited by tail pinch (Zweifel et al., 2011). Studies of combing optogenetics have found that the single-unit activity of DA neurons in mice increased in response to an aversive stimulus (Cohen et al., 2012).

### Nucleus accumbens

The midbrain includes two main neural dopamine pathways, its cortex and limbic system. The former leads from the VTA to the prefrontal cortex and the latter from the VTA to the NAc. The NAc serves as an important downstream target of the VTA in the midbrain limbic system pathway. It receives input from VTA DA neurons and is also an important downstream target of dorsal raphe nucleus (DRN) serotonin neurons (Groenewegen et al., 1999; Ikemoto, 2007; Watabe-Uchida et al., 2012). The NAc comprises the ventral part of the striatum. Most of the cells in the NAc, as in the dorsal striatum, are GABA-containing MSNs, with the remainder being cholinergic and GABAergic inter-neurons (Meredith, 1999). Striatal regions contain subpopulations of these MSNs, the so-called “direct” and “indirect” pathways (Gerfen et al., 1990; Surmeier et al., 2007).

The MSNs of the direct pathway predominantly co-express D1 receptors and the endogenous opioid peptide dynorphin while the MSNs of the indirect pathway predominantly co-express D2 receptors and the endogenous opioid peptide enkephalin (Humphries and Prescott, 2010; Sesack and Grace, 2010). According to traditional explanations, the action of DA on the D1 receptors is coupled with the G-protein (G_s_, stimulatory) and associated with the activation of adenylate cyclase, which tends to excite the MSNs of the direct pathway (Albin et al., 1989b). Elevation in the activity of these cells increases GABAergic and dynorphin input to the mesolimbic system and negative feedback on the midbrain dopamine cells. By contrast, the action of DA on the D2 receptors is coupled with glucose-inhibited (Gi, inhibitory) and associated with the inhibition of the adenylate cyclase, which often inhibits the MSNs of the indirect pathway (Albin et al., 1989a; Surmeier et al., 2007) and reduces the GABAergic input of enkephalin from the VP area. Through multiple synaptic connections, then, the inhibition of the indirect pathway at the level of the NAc, ultimately, activates the thalamus (Kelley, 2004).

The various projection pathways of the MSNs in the striatum indicate that the behavioral results that they guide may be inconsistent when they perform related adjustments of the nervous system. The canonical understanding is that the D1 receptors that express striatal neurons convey information directly to the output nuclei of the basal ganglia whereas D2-expressing neurons transmit information indirectly through pallidal neurons (Yawata et al., 2012; Bock et al., 2013; MacAskill et al., 2014). The NAc D1-MSNs encode rewards whereas D2-MSNs code aversion (Hikida et al., 2010; Lobo et al., 2010; Kravitz et al., 2012; Tai et al., 2012). Studies using mouse models have suggested that the optogenetic stimulation of the D1 receptors in the dorsal striatum induces persistent reinforcement while activation of the D2 receptors induces transient punishment (Kravitz et al., 2010, 2012).

Recent studies, however, have challenged the canonical view. Combining optogenetics and electrophysiology, (Kupchik et al., 2015) found that 50% of the VP cells in mice received input from D1-MSNs. Furthermore, researchers found that the optical stimulation of dyn-expressing cells in discrete subregions of the NAc neural pathway drove opposite motivated behavioral states, meaning that light stimulation of dyn-expressing cells in the vNAcSh drove aversive behavior. Conversely, the stimulation of dyn-expressing cells in the dNAcSh elicited reward behavior. The activation of dynorphin neurons in the ventral part of the NAc in mice overlaps for the most part with D1 receptors, leading to aversive production (Al-Hasani et al., 2015). D1 MSNs projecting to the LH are capable of causing anhedonia and behavioral despair (O’Connor et al., 2015; Massaly et al., 2019; Pignatelli et al., 2021). A mouse model for stress-induced anhedonia and passive coping showed that these phenomena were associated with increases in the synaptic strength of the excitatory synapses in the ventral hippocampus (VH) on D1-MSNs in the NAc medial shell (NAcmSh) and with LH projecting D1-MSN (Pignatelli et al., 2021). Using the mouse model, (O’Connor et al., 2015) found that D1-MSNs provided the dominant source of accumbal inhibition to the LH and rapid control over feeding through the LH GABA neurons. The molecular evidence suggests that mice lacking D2 receptors are less sensitive to the reward effect of cocaine. Combining this finding with evidence that human addicts experience reduced binding of the D2 receptors expressed in the NAc, it appears that the D2 receptors play a crucial role in encoding rewards (Volkow et al., 2007). Furthermore, researchers suggest that D1- and D2-MSNs may exert a concurrent action in reward-related behaviors (Steinberg et al., 2014; Soares-Cunha et al., 2016, 2018; Vicente et al., 2016; Natsubori et al., 2017) selectively activated DA neurons in transgenic rats using genetically targeted tools and demonstrated that NAc D1 and D2 receptors alike contribute to VTA ICSS.

## Role of the basal forebrain in reward and aversion

The BF is a part of the ventral structure of the telencephalon and diencephalon, including the VP, substantial innominate (SI), medial septum (MS), vertical and horizontal diagonal band nuclei (VDB and HDB), magnocellular preoptic nucleus (MCPO), and other regions. It plays a role in cognitive functions such as attention, learning, and motivation (Voytko et al., 1994; Lin and Nicolelis, 2008) and is known to be involved in Alzheimer’s disease and other forms of dementia as well as cognitive aging (Gallagher and Colombo, 1995; Grothe et al., 2012). BF neurons have also been found to be associated with reward (Whalen et al., 1994; Tashakori-Sabzevar and Ward, 2018). In recent years, the function of the BF in the regulation of reward has attracted attention.

### Neural structure of the basal forebrain

The BF contains many types of neurons, the most common being cholinergic, GABAergic, and glutamatergic neurons. There is some variability in the type, morphology, and projection pattern of neurotransmitters across these types of neurons (Henny and Jones, 2008; Záborszky et al., 2018). Neurons in the BF express various types of calcium-binding proteins, including calmodulin, calpain, and parvalbumin (PV), as well as other molecular tags (e.g., neuropeptide-Y) and growth hormone inhibitors [e.g., somatostatin (SOM)] (Zaborszky et al., 2012). Owing to the highly complex structure and composition of this region, the relationship of the numerous types of neurons in this region with the regulatory mechanisms of reward and aversion has not yet been fully established.

Cholinergic neurons account for 20% of BF neurons and mainly project to the neocortical carotid and other cortices (Woolf, 1991). They also co-express with other neurotransmitters such as epinephrine, norepinephrine, DA, GABA, glycine, VGLUT1, VGLUT2, and enkephalin. Cholinergic neurons also promote local cortical state regulation and coordinate the activities of cortical circuits involved in various functions (Záborszky et al., 2018). Given the role of these neurons in cognitive regulation, the central nervous system of elderly populations and patients with dementia can be expected to display obvious cholinergic dysfunction related to memory loss (Bartus et al., 1982). Optogenetic studies have found that nearly all cholinergic neurons show an activation response to the main enhancer, such as a water reward or an air sac punishment (Gu and Yakel, 2011; Gu et al., 2012). In addition, cholinergic neurons transmit strengthening signals to the cerebral cortex that can induce plastic changes in V1 to generate reward signals (Eggermann et al., 2014; Liu, 2015).

In recent years, researchers have identified GABAergic and glutamatergic neurons in the BF, which also play important roles in the neural regulation of the brain (Hassani et al., 2009). Non-cholinergic neurons in the BF are involved in the regulation of behavioral states, such as attention and sleep (Hangya et al., 2015; Xu et al., 2015). Optogenetic stimulation of BF PV neurons in mice can regulate the function of the cortex by inducing GABAergic resonance (Kim et al., 2015). Studies using mouse models have also reported that light-activated cholinergic, glutamatergic, and PV-expressing GABAergic neurons in the BF promote wakefulness, while activated SOM-expressing GABAergic neurons enhance sleep (Xu et al., 2015).

The cholinergic, glutamatergic, SOM, and PV neurons of the BF interact: Glutamatergic neurons have outputs to cholinergic and PV neurons, while SOM neurons project to the other three types of neurons (Xu et al., 2015). Both cholinergic and non-cholinergic neurons in the BF can enhance cortical activity, but cholinergic neurons respond mainly to enhancers (Hangya et al., 2015). However, non-cholinergic neurons provide clear clues for predicting the motivation of the enhancement-phase response (Lin and Nicolelis, 2008; Avila and Lin, 2014) and are related to the operational measures of attention (Hangya et al., 2015). Therefore, cholinergic and non-cholinergic neurons may have complementary and synergistic functions in arousal and cognition. Recent studies using mouse models have found non-cholinergic neurons in the BF to be involved in the regulation of reward (Zhu et al., 2017; Faget et al., 2018), but the specific mechanisms of neural regulation remain unclear.

The BF receives many neuronal inputs from and also projects to other brain regions. Thus, cholinergic neurons from numerous sub-regions project to various brain regions. Those from the HDB and MCPO project mainly to the olfactory bulb and the piriform and internal olfactory cortices. Cholinergic neurons from the MS and VDB project mainly to the hippocampus and other brain regions, while those from the VP and SI project primarily to the basolateral amygdala (BLA) and neocortical brain regions (Zaborszky et al., 2012). Cholinergic neurons in the subregions of the HDB, MCPO, MS, and VDB project to orexin neurons in the LH (Sakurai et al., 2005). In addition, cholinergic neurons in the BF receive inputs from several cortical areas, such as the orbitofrontal, motor, and insular cortices, as well as from brain regions, including the lateral septum, central amygdaloid nucleus (CEA), paraventricular nucleus of the hypothalamus, DRN, and parabrachial nucleus (Gielow and Zaborszky, 2017). In studies of the projections of BF non-cholinergic neurons, researchers have reported that PV, GABAergic, and glutamatergic neurons project to each other in the brain regions that encode reward modulation, including the NAc, VTA, and LH (Zaborszky et al., 2012; Do et al., 2016). SOM neurons in the BF mainly receive input from the LS and NAc and project to the LH, VTA, and LHb (Zhu et al., 2017). However, further investigation is needed to determine whether the mutual projection relationships among these neurons are involved in the neuromodulation of reward and their underlying mechanisms.

Located on the dorsal side of the BF, the VP plays a critical role in processing and executing motivated behaviors in the basal ganglia (Mogenson et al., 1980). It has a wide range of upstreams and downstreams in the brain. Researchers have offered a comprehensive interpretation of the central position of the VP as the “common pathway” of reward processing in the brain (Kalivas and Volkow, 2005; Smith et al., 2009). However, the complexity of the VP in terms of the several types of neurons and their interactions has impeded the study of the mechanism of neural regulation underlying reward and aversion.

### Basal forebrain subregion: Function of the ventral pallidum in the regulation of reward and aversion

#### Upstream and downstream connections in the ventral pallidum

**FIGURE 2:**
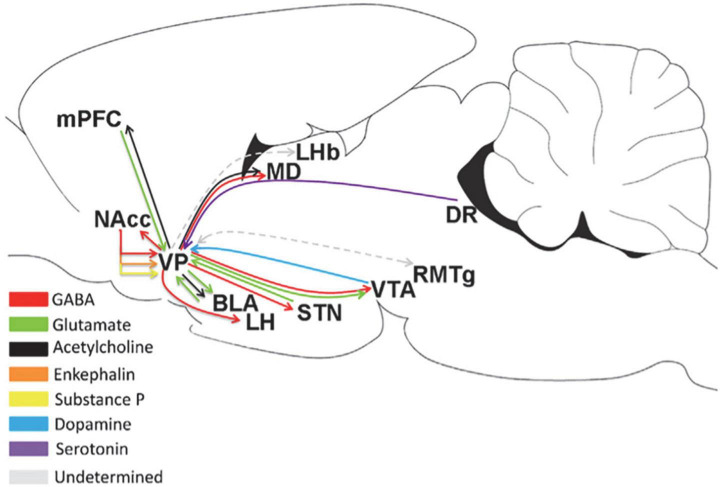
Upstream and downstream projections of the VP in the neural circuits of the brain (Root et al., 2015). MD, medial thalamus; DR, dorsal raphe; STN, subthalamic nucleus.

The VP is the subregion of the BF that receives projections from multiple brain regions, as shown in Figure 2. Scientists have predicted that the VP is an important dopamine-projecting brain region with a regionally oriented dopaminergic input. The lateral VTA is projected to the VPr, VPvm, VPdl, and VPvl, and the midline VTA is projected to the medial part of the VP (Groenewegen et al., 1993; Del-Fava et al., 2007; Taylor et al., 2014). The dopaminergic projections from the VTA to the various neuronal types of the VP include PV-immunoreactive, non-PV-immunoreactive, and cholinergic neurons (Zaborszky, 1989; Gaykema and Zaborszky, 1997). The greatest input to the VP is the inhibitory input from the NAc (Maurice et al., 1998; Usuda et al., 1998). The VP also receives input from the subthalamic nucleus and a small amount of the inferior cortex, including the amygdala. The amygdala is the main glutamatergic input of the VP (Fremeau et al., 1991), and the projection of D1 neurons to the VP is controlled by the input from the amygdala (Napier, 1992), indicating that the excitatory input from the amygdala to the VP may be regulated by D1 receptors. In addition, the VP receives serotonin input from the DRN (Heidenreich and Napier, 2000).

As the “common pathway,” the VP has a wide range of projections to other brain regions, as shown in Figure 2. It has a strong projection to the LHb and a reciprocal projection with the rostromedial tegmental nucleus (RMTg); meanwhile, the RMTg is an input to the LHb, and the downstreams of the RMTg include the VTA, SNc, and DRN (Tripathi et al., 2013). Accordingly, the VP may influence the motivation of behavior through the RMTg. Another important downstream of the VP is the LH. As an important center for monitoring physiological balance in the brain, the LH plays a role in eating. Therefore, the VP may regulate eating through the LH (Tripathi et al., 2013; Zhu et al., 2017). The GABAergic and glutamatergic neurons in the VP project to the VTA (Kalivas et al., 1993; Geisler and Wise, 2008). The VP strongly inhibits the dopaminergic and non-dopaminergic neurons of the VTA through GABAergic release (Hjelmstad et al., 2013). This effect can reduce the number of VTA neurons in the active/projective state (Floresco and Grace, 2003). In addition, the output of the lateral part of the VP spreads laterally from the VTA to the SNc, and these midbrain regions project DA neurons to the dorsal striatum (Haber et al., 2000). VP neurons establish synaptic connections with dopaminergic neurons in both the VTA and SNc (Watabe-Uchida et al., 2012; Ogawa et al., 2014). The projection from the VP to the NAc involves small branch axons with many variations (Haber et al., 1985). The proportion of VPdl and VPdm neurons that project from the VP to the NAc is approximately the same (Spooren et al., 1996; Tripathi et al., 2013). Some PV-immunoreactive neurons in the VP project to the NAc (Kuo and Chang, 1992), indicating that among them, VP neurons project GABAergic neurons. The VP also projects to the BLA (Haber et al., 1985; Mascagni and McDonald, 2009). Approximately 75% of VP neurons projecting to the BLA are cholinergic neurons, and these cholinergic neurons emit a single, non-lateral projection to the BLA (Carlsen et al., 1985; Zaborszky and Cullinan, 1996). Only 30% of cholinergic neurons in the VP co-express VGluT3 mRNA, and 92% project to the BLA, expressing VGluT3 mRNA (Nickerson Poulin et al., 2006).

#### Research progress on the regulation of reward and aversion in the ventral pallidum

The VP is the focal point of the limbic circuit in the brain and is an important convergence point in the neuroregulatory circuits related to motivation and reward in patients with depression (Haber et al., 1985; Smith et al., 2009). It is also related to behaviors such as reward, pleasure, and drug addiction (Tindell et al., 2004).

The VP plays an important role in the reward neural pathways. Early researchers have linked the VP to reward and motivational functions, suggesting that electrical damage to the pallidum causes mice to fail to eat actively and perform water ingestion behaviors (Morgane, 1961). Cromwell and Berridge (1993) found that local lesions in the VP eliminate the “like” performance of individuals. When the VP is affected, individuals lose their positive hedonic response to the taste of delicious food (Cromwell and Berridge, 1993; Berridge, 1996). GABA-A receptor antagonists injected to the VP of mice significantly increased eating behavior and food intake, and disinhibition of GABA in the VP enhanced the mechanism of behavioral or food “wants” in mice. This disinhibition also reduced individual avoidance and aversion to tastes previously associated with lithium chloride (Stratford and Kelley, 1999; Inui et al., 2007; Smith et al., 2009). On the contrary, activation of GABA-A receptors in the VP *via* injection of an agonist (muscimol) decreased the normal pleasure response of individuals to sucrose and enhanced their aversive response (Shimura et al., 2006). In addition, the VP receives a large amount of GABAergic input from the NAc (Chrobak and Napier, 1993). Mice with GABAergic inactivation in the VP are less inclined to work on instrumental tasks to obtain sucrose reward, preferring instead to directly select more readily available food (Farrar et al., 2008). Neuronal inactivation in the VP reduces Pavlovian states of incentive learning for rewards, such as acquiring and expressing acquired preferences for sucrose, amphetamines, and other conditions that are paired with reward (Hiroi et al., 1999; Dallimore et al., 2006).

The VP is also involved in the neural circuits that regulate aversion. Optogenetic studies have found that suppressed PV neurons from the VP projecting to the LHb induced a strong preference, consequently inducing related aversive behaviors and even blocking cocaine-induced preferences (Knowland et al., 2017). The DA levels in the VTA have been found to return to normal levels after injection of a glutamate receptor antagonist to the VP of trained mice; this regulation is likely linked to the BLA–VP–VTA pathway (Chang and Grace, 2014). GABAergic and glutamatergic VP neurons selectively control behavior in opposing motivational contexts. *In vivo* recording combined with optogenetics in mice revealed that these two neurons oppositely encode positive and negative motivational values, are differentially modulated by animals’ internal state, and determine the behavioral response during motivational conflict (Stephenson-Jones et al., 2020). Injecting enkephalins to the VP can increase the “like” performance of animals and induce some defensive behaviors. Additionally, optogenetic activation of VP glutamatergic neurons induces aversion in mice and inhibits their preference for reward (Faget et al., 2018; Tooley et al., 2018).

## Other brain regions

The LHb is considered to be the brain region that classically regulates aversion. It receives afferent information from the basal ganglia and limbic forebrain and mainly projects to the RMTg as well as the midbrain monoaminergic nuclei (Sutherland, 1982; Brinschwitz et al., 2010; Zhou et al., 2017). Optogenetic studies in mouse models have found that stimulating the LHb–VTA pathway can lead to strong conditioned place aversion. Further, the glutamatergic neurons of the LHb project to the DA neuronal synapses of the VTA DA–mPFC. Injection of related antagonists to the mPFC can prevent conditioned place aversion caused by optogenetic stimulation of the LHb–VTA pathway (Lammel et al., 2012). In mouse and rat models, optogenetic stimulation of excitatory afferents to the LHb in brain areas, including the VP, can also induce individual aversion (Yoo et al., 2016; Zhang et al., 2018). In contrast, local injection of ketamine in rats can inhibit the neuronal activity in the LHb and produce antidepressant/anxiolytic effects (Dolzani et al., 2016; Tchenio et al., 2017). The LHb is involved in regulating dopaminergic neurons in the VTA in processing reward information and mediating reward-related behavioral responses (Bromberg-Martin et al., 2010; Stopper and Floresco, 2014). Through its connections with both the dopaminergic and serotonergic systems, the LHb may integrate information on both value state and value change, making it an ideal node to control reward-based decision-making and mood (Nakamura et al., 2008; Proulx et al., 2014). Glutamatergic neurons in the LHb project to the RMTg, regulating reward prediction and learning. During the negative RPE process, LHb neurons are activated and send excitatory signals to RMTg inhibitory neurons, which project to DA neurons and inhibit DA neurons projecting to other brain regions (Matsumoto and Hikosaka, 2007; Kaufling et al., 2009; Lammel et al., 2012). Studies on monkeys and rats found that the LHb regulates the firing of DA neurons during negative RPE signaling and provides indicative signals for reward-seeking behaviors (Bromberg-Martin and Hikosaka, 2011; Zapata et al., 2017).

The amygdala is the subcortical center of the limbic system. It is considered an important brain area for processing emotions, such as fear and anxiety, and is involved in the regulation of reward neural circuits (Winston et al., 2005; Shabel and Janak, 2009). The BLA–NAc and BLA–CEA pathways have been shown to encode positive and negative information differently (Namburi et al., 2015). Optogenetic studies have found that BLA cholecystokinin-positive and -negative neurons encode two opposed emotional experiences, including disgust and pleasure. In real-time place preference experiments, activation of cholecystokinin peptide neurons in the BLA induces mice to produce a photocurrent response. On the contrary, activation of neurons in the BLA that do not express cholecystokinin causes mice to show preference for the illuminated area. Both groups of amygdala neurons project to the NAc, where positive neurons form synaptic connections mainly with inhibitory neurons of D2 receptors in the NAc, while negative neurons form synaptic connections mainly with the inhibitory neurons of D1 receptors (Shen et al., 2019).

The DRN is a major source of serotonin and is a nerve nucleus located in the midbrain (Vertes, 1991; Jacobs and Azmitia, 1992). It has long been considered to be closely related to psychiatric conditions, including anhedonia, anxiety, and depression (Clarke et al., 2004; Crockett et al., 2009). The DRN is also involved in the neural circuits that encode reward. Activation of DRN neurons produces a strong reward effect that provides strong pleasure and motivation to maintain the formation of ICSS and mediates accurate reward learning in mice. Through specific gene knockout mouse strains, both serotonin and glutamatergic neurons are proven to be involved in the expression of reward signals in the DRN, but their functions differ. Glutamatergic neurons provide the basic reward motivation, while serotonin neurons are responsible for maintaining a high level of motivation in more complex and challenging situations (Liu et al., 2014). Researchers used rabies virus tracing, electrophysiology, and behavioral assay to investigate the precise connections between the VTA and DRN in mice and found that the inhibitory GABA neurons of the rostral VTA (rVTA) and caudal VTA projected two different groups of neurons in the DRN. The activation of these two loops had opposite behavioral phenotypes, which produced aversion and reward signals, respectively. Activating the rVTA to the DRN pathway can specifically reduce the reward memory of morphine without affecting its analgesic effect (Li et al., 2019). This may also become an effective means of treating opioid addiction. In addition, DA neurons of the DRN are involved in the neural circuits that control the expression of memory related to reward and punishment. In mouse models, the LPB glutamatergic neuron–DRN DA neuron pathway regulates reward memory. Morphine intake enhances the excitability of neurons in the LPB projecting to the DRN. Inhibition of the LPB–DRN pathway reduces the expression of pleasure-related memories of morphine intake and destroys the encoding of reward information by DA neurons of the DRN (Lin et al., 2021).

## Summary

Recent advances in techniques that facilitate the identification and manipulation of brain circuits have allowed researchers to make great progress in investigating the neuromodulatory mechanisms that encode and process reward and aversive signals. Owing to limited space, this article primarily focuses on the neural mechanisms involved in the regulation of reward and aversion in certain brain regions, including the NAc, VTA, BF, and LH.

A loss-of-function study with temporal precision and cell-type specificity is warranted to clarify the neuroregulatory functions of specific types of neurons in different brain regions in reward. Although a series of optogenetic experiments have demonstrated that activation of specific types of neurons in brain regions, such as the NAc, VTA, and BF, can effectively induce reward effects, including conditioned place preference and motivation enhancements, the experimental demonstration of their necessity is still needed. The use of photosensitized inhibition channels, including NphR or Arch, or activation of local GABA neurons may be feasible. Furthermore, although brain regions such as those discussed in this paper play an important role in the neural circuit regulation of reward and aversion, the specific neural regulatory mechanism of reward and aversion by different types of neurons in these regions is unknown.

While new technologies such as optogenetics have aided the progress of understanding the mechanisms of brain neural activities, there still remain some limitations. Some neurons can release multiple neurotransmitters, which is a complex problem in rodents. Fluorescence *in situ* hybridization (Xiu et al., 2014) may be an effective technique to solve this problem. Target neurons are tagged by fluorescent-labeled AAV virus of Cre transgenic mice and then hybridized with fluorescent-labeled specific DNA or RNA probes. The subtypes of target neurons can be determined by analyzing the fluorescence hybridization signals. In addition, the whole-cell patch-clamp technique has been used as a powerful tool for analyzing local circuits of the central nervous system in a brain slice preparation in which the fundamental architecture of local circuits is mostly maintained. The cell types can be detected by recording the spontaneous neuronal activity of target neurons under current clamping at resting conditions. Viral neuronal tracing combined with the use of Cre-dependent constructs and Cre mouse lines has recently offered a highly selective approach to target neurons. In combination with opto/chemogenetics, calcium imaging, and behavioral analysis, this approach is capable of investigating the role of neurons and mapping the related neural circuits. Integration of different techniques, to some extent, creates a useful means to examine the function of neurons. However, this approach is not suitable for addressing all subtypes of neurons owing to the feasibility of site-specific injection or the presence of sub-subtypes. In this case, the use of transgenic animal models may offer an advantage, yielding the possibility of generating a new line based on the genetic profile of neuronal subtypes.

Optogenetics-based neural tracing technology can output a specific whole-brain mapping that shows unprecedented information. Nevertheless, some neural tracing results indicate that although different types of cells within the same brain area have different or even opposite functions, there is almost no difference in the input brain area and input cell types. Therefore, it would be helpful to develop inputs capable of labeling both cell types (e.g., VTA DA and VTA GABA) using multiple colors in the same mouse brain to investigate whether their input brain regions have specific spatial distribution patterns.

The knowledge about reward and aversion may provide guidance in developing novel treatment strategies for neuropsychiatric diseases. Combined with recent advances in human neuroimaging (Hennigan et al., 2015; Boeke et al., 2017), continued research on the heterogeneity of DA neurons will provide insight into how anatomically and functionally distinct DA subsystems are dysregulated in psychiatric diseases. Furthermore, with continuous application of recently developed methods as they become available in creative and rigorous manners, it is believed that essential advances in the understanding of circuit functions about reward and aversion will be made. Such advances will facilitate a more sophisticated understanding of the many adaptive and pathological behaviors and cognitive functions in which these circuits participate.

## Author contributions

The author confirms being the sole contributor of this work and has approved it for publication.
